# Acinetobacter baumannii NCIMB8209: a Rare Environmental Strain Displaying Extensive Insertion Sequence-Mediated Genome Remodeling Resulting in the Loss of Exposed Cell Structures and Defensive Mechanisms

**DOI:** 10.1128/mSphere.00404-20

**Published:** 2020-07-29

**Authors:** Guillermo D. Repizo, Martín Espariz, Joana L. Seravalle, Juan Ignacio Díaz Miloslavich, Bruno A. Steimbrüch, Howard A. Shuman, Alejandro M. Viale

**Affiliations:** a Instituto de Biologia Molecular y Celular de Rosario (IBR, CONICET), Departamento de Microbiologia, Facultad de Ciencias Bioquimicas y Farmaceuticas, Universidad Nacional de Rosario, Rosario, Argentina; b Department of Microbiology, University of Chicago, Chicago, Illinois, USA; Escola Paulista de Medicina/Universidade Federal de São Paulo

**Keywords:** environmental *Acinetobacter baumannii*, preantibiotic-era *Acinetobacter baumannii*, environmental reservoirs, comparative genomics, insertion sequences, virulence factors

## Abstract

Acinetobacter baumannii represents nowadays an important nosocomial pathogen of poorly defined reservoirs outside the clinical setting. Here, we conducted whole-genome sequencing analysis of the Acinetobacter sp. NCIMB8209 collection strain, isolated in 1943 from the aerobic degradation (retting) of desert guayule shrubs. Strain NCIMB8209 contained a 3.75-Mb chromosome and a plasmid of 134 kb. Phylogenetic analysis based on core genes indicated NCIMB8209 affiliation to A. baumannii, a result supported by the identification of a chromosomal *bla*_OXA-51_-like gene. Seven genomic islands lacking antimicrobial resistance determinants, 5 regions encompassing phage-related genes, and notably, 93 insertion sequences (IS) were found in this genome. NCIMB8209 harbors most genes linked to persistence and virulence described in contemporary A. baumannii clinical strains, but many of the genes encoding components of surface structures are interrupted by IS. Moreover, defense genetic islands against biological aggressors such as type 6 secretion systems or CRISPR-cas are absent from this genome. These findings correlate with a low capacity of NCIMB8209 to form biofilm and pellicle, low motility on semisolid medium, and low virulence toward Galleria mellonella and Caenorhabditis elegans. Searching for catabolic genes and concomitant metabolic assays revealed the ability of NCIMB8209 to grow on a wide range of substances produced by plants, including aromatic acids and defense compounds against external aggressors. All the above features strongly suggest that NCIMB8209 has evolved specific adaptive features to a particular environmental niche. Moreover, they also revealed that the remarkable genetic plasticity identified in contemporary A. baumannii clinical strains represents an intrinsic characteristic of the species.

**IMPORTANCE** Acinetobacter baumannii is an ESKAPE (Enterococcus faecium, Staphylococcus aureus, Klebsiella pneumoniae, Acinetobacter baumannii, Pseudomonas aeruginosa, and *Enterobacter* species) opportunistic pathogen, with poorly defined natural habitats/reservoirs outside the clinical setting. A. baumannii arose from the Acinetobacter calcoaceticus-A. baumannii complex as the result of a population bottleneck, followed by a recent population expansion from a few clinically relevant clones endowed with an arsenal of resistance and virulence genes. Still, the identification of virulence traits and the evolutionary paths leading to a pathogenic lifestyle has remained elusive, and thus, the study of nonclinical (“environmental”) A. baumannii isolates is necessary. We conducted here comparative genomic and virulence studies on A. baumannii NCMBI8209 isolated in 1943 from the microbiota responsible for the decomposition of guayule, and therefore well differentiated both temporally and epidemiologically from the multidrug-resistant strains that are predominant nowadays. Our work provides insights on the adaptive strategies used by A. baumannii to escape from host defenses and may help the adoption of measures aimed to limit its further dissemination.

## Abstract (precis)

Acinetobacter baumannii is an ESKAPE (Enterococcus faecium, Staphylococcus aureus, Klebsiella pneumoniae, Acinetobacter baumannii, Pseudomonas aeruginosa, and *Enterobacter* species) opportunistic pathogen, with poorly defined natural habitats/reservoirs outside the clinical setting. A. baumannii arose from the Acinetobacter calcoaceticus-A. baumannii complex as the result of a population bottleneck, followed by a recent population expansion from a few clinically relevant clones endowed with an arsenal of resistance and virulence genes. Still, the identification of virulence traits and the evolutionary paths leading to a pathogenic lifestyle has remained elusive, and thus, the study of nonclinical (“environmental”) A. baumannii isolates is necessary. We conducted here comparative genomic and virulence studies on A. baumannii NCMBI8209 isolated in 1943 from the microbiota responsible for the decomposition of guayule, and therefore well differentiated both temporally and epidemiologically from the multidrug-resistant strains that are predominant nowadays. Our work provides insights on the adaptive strategies used by A. baumannii to escape from host defenses and may help the adoption of measures aimed to limit its further dissemination.

## INTRODUCTION

The genus Acinetobacter, family *Moraxellaceae*, class *Gammaproteobacteria*, is characterized by Gram-negative aerobic coccobacilli of ubiquitous environmental distribution and large metabolic capabilities (1). In the Acinetobacter genus, the phylogenetically closely related species composing the A. calcoaceticus-A. baumannii complex represent opportunistic pathogens that are important nowadays (2). Infections due to A. baumannii in particular, rarely reported in health care settings before the 1970s, rapidly increased in importance with the global spread of a limited number of epidemic clonal complexes (CCs) possessing multidrug resistance (MDR) phenotypes (2, 3). Strains composing the CCs generally contain a variety of genetic material obtained by horizontal gene transfer such as plasmids and chromosomally located genomic islands (GIs), including resistance islands (RIs) encompassing different transposons and integrons which play pivotal roles in both antimicrobial and heavy metal resistances (2, 4–6). CC strains also carry a large repertoire of insertion sequences (ISs) capable of mediating gene inactivation as well as genome rearrangements, including deletions and inversions with strong adaptive significances (2, 6–8). The combination of the above factors, added to the intrinsic resistance of A. baumannii to desiccation and fever-associated temperatures (9), are considered main factors of persistence of this pathogen in the nosocomial environment (2, 7).

Whole-genome sequence (WGS) comparisons have become commonplace in examining strain-to-strain variability and in comparing pathogenic strains with environmental relatives of a given species, in efforts to identify underlying genetic determinants and mechanisms responsible for phenotypic dissimilarities. When applied to A. baumannii, these approaches provided valuable information on the clinical population structure of this species, its potential virulence factors, and the origins and acquisition of antimicrobial resistance determinants (2, 4, 6, 7, 10, 11). However, the identification of virulence traits both within and between CC members has remained elusive, suggesting a complex and even multifactorial nature of mechanisms involved (2, 3, 7). The importance of a deeper genomic study of nonclinical (“environmental”) isolates has therefore recently been emphasized to clarify both the virulence potential of the aboriginal A. baumannii population and the evolutionary paths that led toward a pathogenic lifestyle (2). Nevertheless, the natural habitats and potential reservoirs of A. baumannii outside the clinical setting have remained poorly defined, and they are only beginning to be elucidated (2, 11–13).

We have recently traced the origins of the collection strain A. baumannii DSM30011 (11) to an isolate originally classified as Achromobacter lacticum, which was obtained prior to 1944 from the enriched microbiota responsible for the aerobic decomposition of the resinous desert shrub guayule (14, 15). WGS of this strain and subsequent comparative analysis with 32 other complete clinical A. baumannii genomes revealed the presence of 12 unique accessory chromosomal regions in strain DSM30011, including five regions containing phage-related genes, five regions encoding toxins related to the type 6 secretion system, and one region encompassing a novel CRISPR-cas cluster (11). As expected from an environmental isolate obtained before the massive introduction of antimicrobial therapy in the 1950s (2), DSM30011 showed a general antimicrobial susceptibility phenotype and no RI in its genome (11). Notably, the genome of DSM30011 lacked complete IS elements, although some remnants could be predicted on it. Remarkably still, most genes and regulatory mechanisms linked to persistence and virulence in pathogenic Acinetobacter species were identified in the DSM30011 genome. Moreover, several gene clusters encoding components of catabolic pathways related to the degradation of plant defenses were found, thus suggesting that plants may provide an effective niche for A. baumannii (11), as reported for other species of the genus (11). In turn, it also opens the possibility that phytophagous insects feeding on these plants (16–22) may represent both reservoirs and vectors for the dissemination of A. baumannii in the environment.

During the original isolation of DSM30011, two companion isolates also classified as A. lacticum displaying similar phenotypic characteristics were also described (14). Only one of the isolates was saved in collections, and we obtained a replica of this isolate (see Table S1; all supplemental files for this paper can be found at https://www.researchgate.net/publication/342762639_Acinetobacter_baumannii_NCIMB8209_a_rare_environmental_strain_displaying_extensive_insertion_sequence-mediated_genome_remodeling_resulting_in_the_loss_of_exposed_cell_structures_and_defensive_mechanis) which had been deposited on the National Collection of Industrial Food and Marine Bacteria, Aberdeen, Scotland under the strain designation NCIMB8209, aiming to perform WGS comparative studies with other completely sequenced Acinetobacter genomes. We report here the results of these analyses as well as some phenotypic characteristics of this strain, which may provide clues into the environmental reservoirs, genomic diversity, and virulence potential of the preantibiotic era A. baumannii population.

## RESULTS AND DISCUSSION

### Phylogenetic analysis assigned strain NCIMB8209 to A. baumannii, albeit to a separate clonal lineage compared to its companion strain DSM30011. (i) NCIMB8209 origins.

As described in our previous work (11), strain NCIMB8209 and its companion strain DSM30011 were isolated prior to 1944 from the natural microbiota enriched during the aerobic decomposition of guayule, an industrial procedure (retting) designed to reduce the resinous content of the shrub processed material for the subsequent production of natural latex (14, 15). Our maximum likelihood (ML) phylogenetic analyses based on core gene sequence comparisons derived from the WGS data (see below) allowed us to confidently assign this strain to A. baumannii as a species. In concordance with this assignment, this strain was capable of growing at 44°C (11) in what represents a typical phenotype associated with A. baumannii (1, 23). Still, random amplification PCR (11), phylogenetic, comparative genome analysis, and metabolic studies (see below) indicated significant differences between this strain and its companion A. baumannii strain DSM30011. This evidence suggests that, even though these two A. baumannii strains might have similar environmental niches, they still belong to separate clonal lineages. NCIMB8209 thus provided us with a different A. baumannii strain isolated from a nonclinical source (14) before the massive introduction of antibiotics to treat infections (2).

### (ii) NCIMB8209 genomic features.

Genome sequencing indicated that the NCIMB8209 chromosome consisted of 3,751,581 bp in length with a G+C content of 39.1% (Table 1). These values match the average values reported for the genomes of the species composing the Acinetobacter genus (3,870 kbp and 39.6%, respectively) (6). It is noteworthy that 223 of the coding DNA sequences (CDSs) (i.e., around 6% of the total) predicted in the NCIMB8209 genome were pseudogenes. It is worth noting that a similarly high number of nonfunctional genes (272; 9% of the total genes) has been reported for the A. baumannii strain SDF, which was isolated from a human body louse and whose genome is riddled with numerous prophages and ISs (24).

**TABLE 1:** General features obtained from NCIMB8209 genomic sequencing

Genomic parameter^*a*^	Value for parameter
Chromosome	
Accession no.	CP028138
Estimated size (bp)	3,751,581
Average GC content (%)	39.1
No. of CDSs	3,596
No. of tRNA genes	73
No. of rRNA genes	18
No. of ncRNA genes	4
No. of phage regions	5
No. of IS copies	79
Plasmid	
Accession no.	CP028139
Estimated size (bp)	133,709
Average GC content (%)	40.1
No. of CDSs	156
No. of tRNA genes	1
No. of rRNA genes	
No. of ncRNA genes	
No. of phage regions	2
No. of IS copies	14

a Based on NCBI Prokaryotic genomic annotation pipeline. ncRNA, noncoding RNA.

Our analysis also showed the presence of a large plasmid of 133,709 bp with a G+C content of 40.1%, hereafter designated pAbNCIMB8209_134 (Table 1). Comparison of pAbNCIMB8209_134 with other plasmids deposited in databases indicated extensive sequence identity with a group of A. baumannii plasmids with a length of more than 100 kb, including pABTJ2 (25). All of these plasmids share a previously undescribed Rep-3 superfamily (pfam0151) replication initiation protein gene (C4X49_18465). The presence of both partition (C4X49_18550) and toxin/antitoxin genes (C4X49_18715 to C4X49_18720) related to plasmid stability were detected in pAbNCIMB8209_134. In contrast, genes involved in mobilization, conjugation, antimicrobial resistance or virulence functions could not be identified in pAbNCIMB8209_134. Still, this plasmid encodes functions which may provide some adaptive advantages to their Acinetobacter hosts, such as a putative glutathione-dependent pathway of formaldehyde detoxification (C4X49_18640 to -18650).

### (iii) Comparisons of the chromosomal architectures of A. baumannii environmental strains NCIMB8209 and DSM30011.

**FIG 1:**
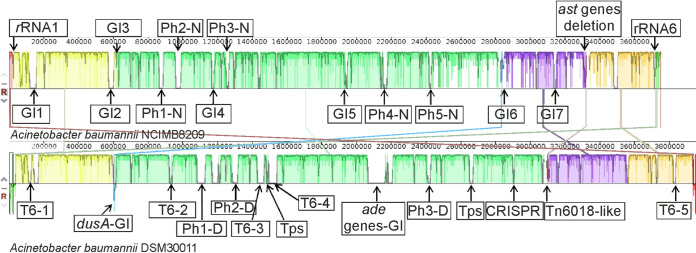
Linear comparison of the genomes of A. baumannii strains NCIMB8209 and DSM30011 inferred using Mauve. Each block corresponds to a DNA fragment of the chromosome distinctively colored for clarity. The degree of conservation is indicated by the vertical bars inside the blocks. Their position relative to the genome line denotes colinear and inverted regions. For a better appreciation of genome synteny, DSM30011 chromosomal DNA shown corresponds to the reverse complementary strand. Putative prophage (PhX-N for NCIMB8209 and PhX-D for DSM30011) and genomic island (GI) insertion sites are indicated (see Tables 2 and 3 for details). Two out of the six rRNA-encoding operons in strain NCIMB8209 are also represented (rRNA1 and rRNA6). Regions encoding interbacterial competition islands (T6-1 to T6-5 and Tps), heavy metals (*dusA*-GI and Tn*6018*-like) and antimicrobial (*ade*-GI) resistance islands and a CRISPR-cas cluster are indicated for DSM30011 genome.

Comparison of the overall chromosome structures of the A. baumannii NCIMB8209 and DSM30011 strains showed that the former is 198 kbp smaller than that of DSM30011 (Fig. 1). Furthermore, a number of GIs and prophages distinguished these two chromosomes as will be described in greater detail below. Despite these differences, a general shared synteny was observed between the two chromosomes, with the notable exception of an inversion of a 53.1-kb region located between the first and sixth rRNA operons (Fig. 1). A similar situation was found in the community-acquired strain A. baumannii D1279779 (26), in which the same region was inverted compared to DSM30011 and to most other clinical strains, including the type strain ATCC 17978 (see Fig. S1 at the URL given above). This rearrangement was probably mediated by homologous recombination between two oppositely oriented rRNA operons bordering this region (26). Still, and although this inversion reverses the orientation of a number of critical housekeeping genes as well as the origin of chromosomal replication (*oriC*), it has no substantial effects on the growth rate of NCIMB8209 compared to DSM30011 in either rich or minimal medium (data not shown).

### (iv) Phylogenomic and MLST analyses.

**FIG 2:**
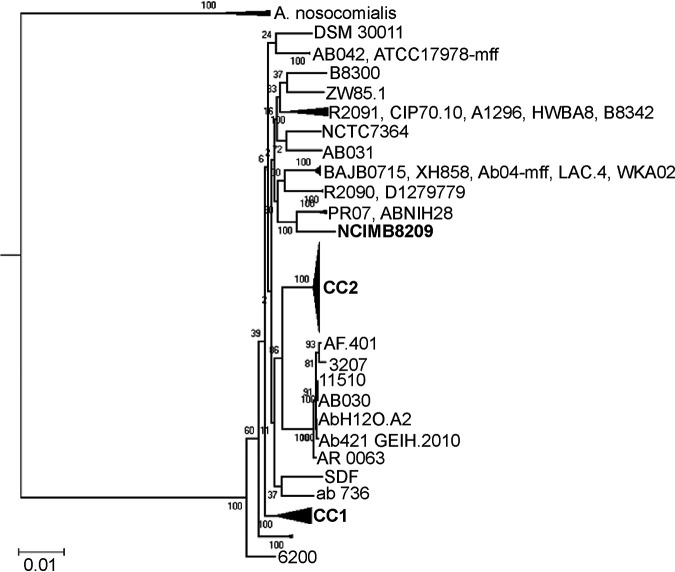
Maximum likelihood phylogenetic analysis of A. baumannii strains. (A) The ML phylogeny was computed based on 383 concatenated core gene sequences (the full tree is shown in Fig. S2). Numbers at nodes correspond to bootstrap values (BV) (100 replicates of the original data set). For simplicity, subnodes for which internal branches showed BV = 100 were collapsed. The scale bar below corresponds to evolutionary distance (average number of the substitutions per site). CC1 and CC2 correspond to subclusters formed by A. baumannii species assigned to epidemic clonal complexes CC1 and CC2, respectively.

A phylogenetic study based on the comparisons of the concatenated sequences of 383 core genes of the environmental strains NCIMB8209 and DSM30011 and a number of Acinetobacter genomes that encompassed 99 other A. baumannii strains as well as 26 non-A. baumannii representatives (26 strains; Table S1) reinforced the affiliation of NCIMB8209 to A. baumannii as a species (Fig. 2 and Fig. S2). Different authors have noted the lack of a defined phylogenetic structure for the general A. baumannii population on phylogenetic trees based on core gene comparisons, with the exception of different terminal clusters each corresponding to an epidemic CC (2, 3, 11, 27). The incorporation of NCIMB8209 core genome sequences to this phylogenetic study did not change this general picture (Fig. 2), but some observations derived from this analysis are worth noting. First, strains NCIMB8209 and DSM30011, which were isolated more than 70 years ago, neither emerged close to the root nor formed a separate “environmental” cluster in the A. baumannii subtree. In contrast, they appeared intermixed between more contemporary clinical strains (Fig. 2). Second, NCIMB8209 emerged in a compact cluster (100% bootstrap support) with a number of recently isolated A. baumannii strains that included PR07 (isolated in 2012 in Malaysia from the blood of a hospitalized patient; NCBI BioProject accession no. PRJNA185400, direct submission) and ABNIH28, a MDR strain isolated in 2016 in the United States from a hospital closet drain (28). Further analyses aiming to compare strain NCIMB8209 with the phylogenetically closest strains ABNIH28 and PR07 (Table S1, ABNIH28-PR07) were performed. We additionally included representative strains of the CCI (ACICU), CCII (AYE), and the NCIMB8209 environmental companion strain DSM30011. Different calculations reflecting evolutionary distance, such as average nucleotide identity (ANI) values, genomic distance, and core-genomic distance shown in this table, reinforced the inferred phylogenetic proximity among the strains included in the subclade under study (Fig. 2). *In silico* DNA-DNA hybridization values were also in line with the calculated values obtained for evolutionary distances. Regarding common gene families, the observed percentages ranged from 76 to 84% for all strains included in the analysis, whereas for additional and absent gene families, these values ranged between 16 and 24% and 18 and 21%, respectively. These nonshared genes, which are part of the accessory genome in each strain, may contribute to the adaption of each particular strain to a particular niche.

Strain NCIMB8209 was assigned to a novel sequence type (ST) (1197 [Table S1]) in the Pasteur scheme of A. baumannii multilocus sequence type (MLST) classification (3). Notably, and in sharp contrast to strain DSM30011, which shared only four of the seven alleles with its closest matching isolate (11), NCIMB8209 shared six of the seven alleles with its closest matching isolates with all differences being restricted to the *rplB* allele (Table S1). Of note, the *gdhB* gene used in the A. baumannii MLST Oxford classification scheme (29) is missing from the NCIMB8209 genome (Table S1), an observation experimentally corroborated by a specific PCR assay (see Materials and Methods for details). A deeper comparative analysis with other A. baumannii genomes indicated that NCIMB8209 lacks a 4.8-kbp region between genes C4X49_10220 and C4X49_10225 which, in other genomes (DSM30011 included), harbors the *gdhB* gene. In the DSM30011 genome, the *gdhB* gene is flanked by a hypothetical gene (DSM30011_07590) and a gene encoding a universal stress protein (DSM30011_07600), and a similar situation occurs in other A. baumannii genomes. This observation indicated that *gdhB* is nonessential for A. baumannii, and in this context, its interruption by IS elements has been previously noted in a number of A. baumannii isolates of clinical origin (30). Moreover, a recent analysis performed with 730 Acinetobacter genomes revealed in 76% of them a paralogous *gdhB* gene (*gdhB2*) in a different locus, the overall observations indicating that the Pasteur MLST scheme presents some advantages for the characterization of epidemiologically related A. baumannii isolates (31).

### (v) NCIM8209 comparative genomics.

A comparative genomic analysis that included the previously mentioned 127 different Acinetobacter sp. strains (Table S1) indicated that 510 representatives of the protein families detected in the NCIMB8209 genome (see Materials and Methods) are present in all of these genomes (Acinetobacter core genome), and 1,149 of them are present in all 101 A. baumannii genomes analyzed (A. baumannii core genome). These numbers differ from estimations obtained by other authors (6) of the core genome content of the Acinetobacter genus (950 genes; 133 genomes analyzed) and A. baumannii in particular (1,590 genes; 34 genomes analyzed). It is worth remarking that even when similar numbers of total genomes were used in both studies, Touchon and collaborators (6) used mostly non-*baumannii* strains, whereas in our study most of the strains corresponded to A. baumannii. This difference and the inclusion of two bona fide A. baumannii environmental strains in our calculations might explain why the A. baumannii core genome estimation was reduced by 27.7% in our study. In this context, recent estimations conducted by other authors (32) with 78 A. baumannii genomes, which included the environmental strain DS002, inferred a core genome of 1,344 genes for this species. The inclusion of more environmental strains in these calculations will certainly contribute to obtain a more accurate value for the core genes repertoire of the species.

### NCIMB8209 antimicrobial resistance.

Conventional antimicrobial susceptibility assays indicated that strain NCIMB8209 showed susceptibility to most clinically employed antimicrobials tested except nitrofurantoin and, among β-lactams, to ampicillin at MIC values just above the CLSI recommended breakpoints (Table S2). In full concordance (Table 2 and Table S2), this strain lacks AbaR resistance islands. The marginal ampicillin resistance of this strain (see above) most likely reflects the presence of a number of β-lactamase genes (Table S2). Of these genes, it is worth mentioning C4X49_08235 (*bla*_OXA-78_) encoding an OXA-51-type carbapenemase with 100% identity to the class D β-lactamase OXA-78 (NCBI WP_005139262.1), and C4X49_12575 (*bla*_ADC-154_) encoding an enzyme identical to the class C ADC-154 β-lactamase (WP_005138362). The presence of these genes in the chromosome of NCIMB8209 reinforces proposals that they provided for the intrinsic β-lactamase gene repertoire of A. baumannii (7, 33–35). NCIMB8209 also contains a *carO* gene (C4X49_13345) encoding a *carO* variant II allele described so far only among the A. baumannii clinical population (36), and also an *oprD/occAB1* homolog (C4X49_01130). These genes encode different outer membrane proteins proposed to participate in the permeation of carbapenems into the periplasm (37). NCIMB8209 also carries genes encoding enzymes providing resistance to aminoglycosides and chloramphenicol including *ant(3”)-II* and *catB*, respectively (Table S2). This indicates both the potentiality to evolve such resistances under selective pressure and an environmental reservoir of these resistance genes.

**TABLE 2:** Genomic islands identified in the chromosome of strain NCIMB8209^*a*^

Region	Length (kbp)	Location	Integrase gene	Flanking genes (accession no.)	Description
GI1	39.7	116596–156278	C4X49_00725	LysR-family transcriptional regulator (C4X49_00535)/tRNA-Gly-GCC (C4X49_00740)	Type III restriction modification system. TA systems (HypBA, YefM-YoeB). ISs
GI2	30.5	569359–599853	C4X49_02720	Hypothetical protein (C4X49_02715)/ peptide chain release factor 3 (C4X49_02900)	Phage proteins. Type I restriction modification system
GI3	19.1	613054–632163	C4X49_02950	*dusA* (C4X49_02945)/MFS transporter (C4X49_03050)	Arsenic and heavy metal resistance island
GI4	12.6	1165713–1178263	C4X49_05820	tRNA-Ser-TGA (C4X49_05765)/ hypothetical protein (C4X49_05860)	Hypothetical proteins of unknown function
GI5	19.1	1927268–1946350	C4X49_09525	Hypothetical protein (C4X49_09520)/ TetR-AcrR family transcriptional regulator (C4X49_09610)	Fatty acid catabolic pathway
GI6	29.3	2818151–2847423	C4X49_13865	Peptide synthetase (C4X49_13745)/ tRNA-Ser-TGA (C4X49_13870)	Heavy metal resistance island
GI7	13.3	3133811–3147160	C4X49_15320	Sensor histidine kinase (C4X49_15265)/ tRNA-Leu-CAA (C4X49_15325)	Type I restriction modification system

a According to NCBI and RAST annotations.

Strain NCIMB8209 shares with DSM30011 (11) susceptibility to folate pathway inhibitors such as sulfamethoxazole-trimethoprim (Table S2). These susceptibilities correlate with the absence of *sul* or *dfrA* resistance genes in the genome (Table S2) and represent a notable exception compared to most clinical strains of A. baumannii, including strains more contemporary to NCIMB8209 such as ATCC 19606 and ATCC 17978 (4, 7, 38–41). This characteristic is compatible with the original isolation of these two strains in an environment still free of the selection pressure derived from the use of sulfonamides common to the clinical setting at that time (11).

Genes encoding components of the RND (resistance-nodulation-cell division), DMT (drug/metabolite transporter), MATE (multidrug and toxic efflux), MFS (major facilitator superfamily), and SMR (small multidrug resistance) efflux systems involved in clinical Acinetobacter strains in the extrusion of toxic compounds, including some antimicrobials (7) were also found in the NCIMB8209 genome (Table S2). Remarkably, a region present in most A. baumannii genomes encoding phage-related proteins and the AdeRS two-component regulatory system and associated AdeABC efflux components (Fig. 2) was missing in the NCIMB8209 genome. A genomic island (GI5) was instead found in an equivalent genomic location (Table 2). The loss of this gene cluster has also been documented in a number of other A. baumannii strains, irrespective of their clinical or environmental origins (10, 31).

### A. baumannii NCIMB8209 shows reduced virulence toward Galleria mellonella and Caenorhabditis elegans.

**FIG 3:**
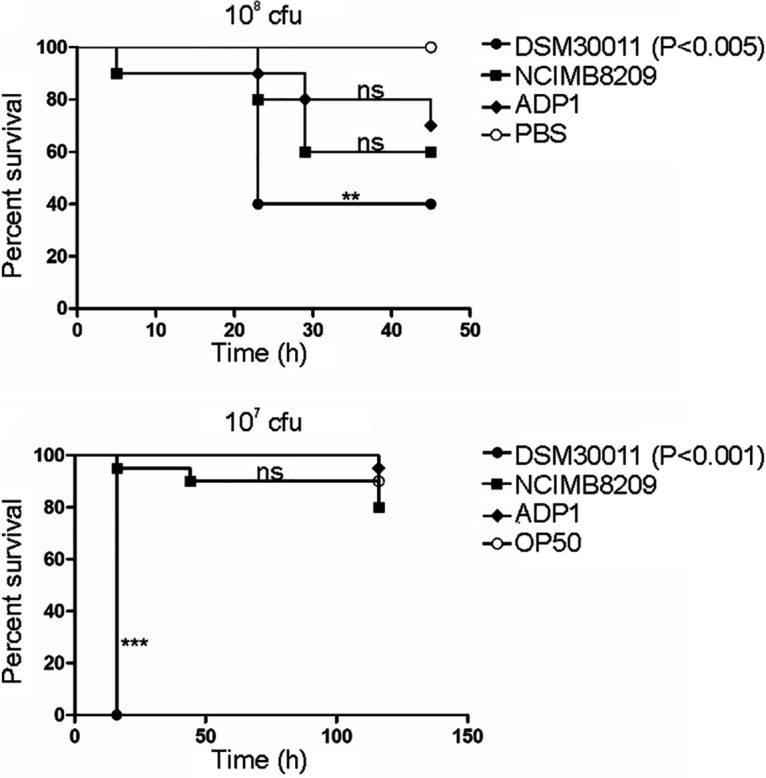
G. mellonella (top) and C. elegans (bottom) lethality curves. Comparative survival analysis between A. baumannii DSM30011 and NCIMB8209 and A. baylyi ADP1 strains. PBS was used as control. (Top) 10^8^ CFU were used. (Bottom) 10^7^ CFU were used. Data are representative of three separate survival experiments, each performed with 20 larvae/worms. Survival curves were constructed by the Kaplan-Meier method and compared by log rank analysis. ns, not significant. **, *P* < 0.01; ***, *P* < 0.005.

G. mellonella moth larvae and the nematode C. elegans provide reliable models to study the virulence of numerous human pathogens, among them Acinetobacter genus species (42, 43). We thus decided to use these two models to evaluate the virulence of strain NCIMB8209 compared to those of strain DSM30011 and the soil organism Acinetobacter baylyi ADP1 (44). In the G. mellonella model, DSM30011 showed high virulence (45, 46), whereas a low-virulence capacity has been demonstrated for strain ADP1 (47). In contrast, virulence in the C. elegans model has not been assessed before for this group of strains. As observed in Fig. 3, NCIMB8209 was less virulent than DSM30011 in either of these models, with the latter environmental strain showing in particular much higher capacity to kill C. elegans. In contrast, NCIMB8209 killing capacity was close to that observed for A. baylyi ADP1 in either assay (Fig. 3). The observed differences in virulence between NCIMB8209 and DSM30011 indicate relevant phenotypic differences between them (see also following sections), regardless of their isolation as companion strains from a similar environmental origin following similar enrichment and culture protocols (14).

### ISs and prophages have extensively modified the NCIMB8209 genome.

The lower virulence displayed by strain NCIMB8209 compared to that of DSM30011 (see above) led us to analyze in more detail their genomes, aiming to find genetic differences that might explain these distinct phenotypes. When comparing the accessory genomes of DSM30011 and NCIMB8209, some differences worth noting were observed in the latter (Fig. 1) such as: (i) the presence of seven GIs (Table 2), which will be described throughout the text; (ii) the presence of five regions harboring putative prophages (Table 1 and Table S3), which will also be described in detail below; (iii) the notorious absence of a CRISPR-*cas* gene cluster common to DSM30011 and other A. baumannii strains (11, 48); (iv) the lack of interbacterial competition islands (ICI), such as those encoding the type 6 secretion system components and/or its associated toxins (45); (v) significant differences regarding the number of ISs and composition between these two strains (see below).

### (i) Prophage content.

Recent bioinformatic analyses uncovered a wide distribution and diversity of prophage contents in A. baumannii (49), with some of the prophages contributing to horizontal gene transfer between strains by generalized transduction (50). Our bioinformatic analysis of phage sequences in the NCIMB8209 genome indicated five regions (Ph1-N to Ph5-N) encompassing phage-related genes (Table 3). According to PHASTER integrity predictions, two “questionable” prophages (Ph1-N and Ph2-N) and three “incomplete” prophages (Ph3-5-N; Table S3) were identified among them. The integration sites for Ph4-N and Ph5-N in the NCIMB8209 genome were the same hot spots found in other A. baumannii genomes (10), whereas those of Ph1-N to Ph3-N represent novel integration sites (Table 3). Of note, both Ph1-N and Ph5-N sequences share significant identity with the podoviral lytic bacteriophage YMC/09/02/B1251 ABA BP (GenBank NC_019541.1) (51), while Ph2-N, Ph3-N, and Ph4-N showed homology with the Acinetobacter phage vB_AbasS_TRS1 (NC_031098) (52). It is also worth noting that the prophages found in NCBIM8209 shared only limited homology with those predicted for DSM30011 (11).

**TABLE 3:** Prophages inserted into the chromosome of strain NCIMB8209^*a*^

Region	Length (kbp)	No. of predicted proteins	Genome position	GC (%)	Flanking genes (accession no.)
Ph1-N	25.8	35	860213–886091	40.3	*smpB* (C4X49_04195)/phosphopantetheine adenylyltransferase (C4X49_04375)
Ph2-N	28.4	41	955316– 983747	37.7	*ssrA* (tmRNA; C4X49_04705)/DUF927 domain- containing protein (C4X49_04925)
Ph3-N	25.2	29	1240267–1265538	41.5	Aspartate kinase (C4X49_06155)/alanine-tRNA ligase (C4X49_06310)
Ph4-N	39.1	32	2140199–2179329	40.5	MFS transporter (C4X49_10600)/hypothetical protein (C4X49_10635)
Ph5-N	13.9	11	2414964–2428883	37.7	*gspL* (C4X49_11920)/phosphohistidine phosphatase SixA (C4X49_12010)

a According to PHASTER predictions. tmRNA, transfer-messenger RNA.

### (ii) IS content.

IS elements are important drivers of genome evolution in A. baumannii and other pathogens, modulating antibiotic resistance gene expression, mediating genome rearrangements, including deletions of substantial chromosomal regions, promoting insertional gene inactivation, etc. (53). Notably, and in sharp contrast to A. baumannii DSM30011 (11), a high representation of transposable genetic elements such as ISs or transposons was found in the genome of strain NCIMB8209. IS*Saga* predictions (54) (Table S3) followed by manual examination corroborated the presence of 12 different ISs (a total of 79 IS copies) in the NCIMB8209 chromosome and 9 different ISs (a total of 14 IS copies) in the plasmid that this strain harbors (Table 4). These values are significantly higher than the estimated average of 33 IS copies per A. baumannii genome determined by Adams and collaborators (8). Moreover, the diversity of IS families (16 total) found in the NCIMB8209 genome is also remarkable when considering that only 7 out of 976 A. baumannii genomes were found to carry 10 or more different IS elements (8). Although most of the IS elements identified in NCIMB8209 were previously reported in different species of the Acinetobacter genus, this strain contains a very particular IS profile for an A. baumannii strain, with two of the most frequent IS elements (IS*Aha2* and IS*Aha3*) originally detected in *A. haemolyticus* (Table 4). Furthermore, as also seen in Table 4, NCIMB8209 harbors two of the five most represented ISs in A. baumannii (8), namely, IS*Aba26* (15 copies) and IS*Aba13* (14 copies). The absence of IS*Aba1* copies in NCIMB8209 is also remarkable, considering the large impact of this IS among clinical A. baumannii genomes (8). Of note, the three most represented chromosomal ISs (IS*Aha3*, IS*Aba26,* and IS*Aba13*) are also carried by plasmid pAbNCIMB8209, suggesting that this plasmid was the vehicle enabling their acquisition.

**TABLE 4:** IS content^*a*^

IS name	Origin	No. of DNA molecules	
		Chromosome^*b*^	pAbNCIMB8209
IS*Aha3*	A. haemolyticus	18 (2)	1
IS*Aba26*	A. baumannii	15 (4)	2
IS*Aba13*	A. baumannii	14 (7)	3
IS*Aba44*	A. baumannii	11 (2)	
IS*Aha2*	*A. haemolyticus*	7 (2)	
IS*1006*	A. junnii	4	
IS*Aba45*	A. baumannii	3	
IS*17*	*A. haemolyticus*	2 (1)	
IS*1008*	A. calcoaceticus	2 (1)	
IS*Aba22*	A. baumannii	1 (1)	2
IS*Aba4*	A. baumannii	1	
IS*Aba31*	A. baumannii	1	2
IS*Aba2*	A. baumannii		1
IS*Aba46*	A. baumannii		1
IS*1007*	Acinetobacter sp.		1
IS*Acsp3*	Acinetobacter sp.		1

a According to ISSaga and ISFinder predictions.

b The numbers of gene-interrupting IS copies are indicated in parentheses.

From the detected ISs in the NCIMB8209 genome, three represented novel elements that were deposited in the ISSaga database (54) under the names IS*Aba44* (IS*630* family), IS*Aba45* (IS*3* family), and IS*Aba46* (IS*66* family). From these elements, IS*Aba44* is present in 11 copies in the NCIMB8209 chromosome (Table 4). This particular mobile element was also found in the chromosomes of three other A. baumannii strains (ABNIH28, B8300, and B8342) and in A. johnsonnii XBB1 (75% identity, query coverage of 100%). No homologs to this IS were found in other organisms outside the Acinetobacter genus. IS*Aba45* (three copies) was also detected in four other A. baumannii strains (PR07, ABNIH28, A1296, and IOMTU433, the former two strains closely linked to NCIMB8209, Fig. 2) and in A. soli GFJ2 (87% identity, query coverage of 99%). Moreover, IS*Aba45* displays significant nucleotide identity with similar ISs present in other Acinetobacter strains (76%) and in Moraxella osloensis plasmids (70%). The IS*Aba46*-only copy is carried by plasmid pAbNCIMB8209, and it was also found in the chromosomes and plasmids of numerous strains of A. pittii, A. baumannii, A. lwoffii, A. junii, A. johnsonnii, and A. haemolyticus (>86% identity, query coverage of 100%). Concerning the locations of the above IS elements, we found that 17 of them are interrupting an equivalent number of CDSs, very likely precluding their expression (Table S3). Seven of these 17 CDSs are interrupted by IS*Aba13* copies (Table 4). Remarkably, a similar situation was reported previously for strain D1279779, which harbors 18 IS*Aba13* copies (26). We also detected that some IS elements were positioned on one of the borders of several genomic islands (GIs), including GI1, GI2, GI4, and GI6 (Table 2). It is tempting to speculate that these insertions probably interfere with the excision mechanism of the corresponding GIs and that they were thus selected due to the subsequent retention of these GIs in the chromosome (55).

In summary, the plethora of IS elements found in strain NCIMB8209 have significantly helped remodel the genome of this strain.

### A genome devoid of interbacterial competition islands.

As mentioned above, the genome of NCIMB8209 carries neither a type 6 secretion system (T6SS) main gene cluster nor any T6SS-associated locus encoding VgrG-like proteins or their cognate toxins (45, 56). Searching for other competition mechanisms such as the two-partner systems (Tps) already identified in strain DSM30011 (Fig. 2) related to contact-dependent inhibition (CDI) (57) also resulted in negative results. These observations led us to hypothesize that the ability of strain NCIMB8209 to outcompete other bacteria was severely compromised. To test this prediction, bacterial competition assays were performed essentially following previously described procedures (45). Results shown in Fig. S3 demonstrate that strain NCIMB8209 was not capable of outcompeting Escherichia coli when the former strain was used as the attacker and E. coli DH5α was the prey. In contrast, the DSM30011 strain was clearly capable of outcompeting E. coli DH5α in a similar assay, an effect that specifically depended on the T6SS system as shown by the lack of effect observed in a DSM30011 Δ*tssM* mutant (Fig. S3A). In correlation with these results, NCIMB8209, similarly to DSM30011 Δ*tssM*, did not secrete detectable amounts of Hcp (a marker of a functional T6SS) into the growth medium (Fig. S3B). We additionally tested the capacity of NCIMB8209 and DSM30011 to outcompete each other. While DSM30011 completely eliminated NCIMB8209 (Fig. S3C) when coincubated in a 10:1 attacker/prey ratio, the latter strain was not capable of outcompeting the former under similar experimental conditions (Fig. S3D).

### Persistence and virulence.

To analyze the presence of genes potentially involved in persistence and virulence in strain NCIMB8209, we performed a BLASTN homology search using as the query a list of potential candidates described in Acinetobacter strains (11). This list included genes coding for the synthesis of the capsule and other exopolysaccharides, appendages, outer membrane (OM) proteins, and the T2SS, genes encoding phospholipases and proteases, and genes involved in traits such as motility and iron scavenging. Our searching indicated that 131 out of the 146 genes (sequence identity of ≥76%) encoding potential virulence factors analyzed were present in NCIMB8209 (Table S2). Among the 15 putative virulence factors included in the search and not detected in the NCIMB8209 genome, most of them encode or are involved in the synthesis of surface-exposed molecules (Table S2). These included the Prp pilus (58), the MFS transporter Pmt (probably involved in DNA transport necessary for biofilm formation [59]), and a surface motility-associated molecule (60). Moreover, the gene encoding the pilus 3 fimbrial adhesion precursor (C4X49_08175), and the pilus 3 fimbria-anchoring protein (C4X49_08180) are both incomplete, and a gene coding for a polymorphic toxin of the RTX family (61) was interrupted by an IS*Aba44* copy (C4X49_17180 to C4X49_17185). We also noticed that several genes whose products are directly or indirectly involved in functions related to adhesion or biofilm formation in clinical strains were absent or interrupted by ISs. For instance, the gene encoding the Bap protein (62) could not be detected (Table S2), and instead an IS*Aba13* copy was identified in the locus encompassing the *bap* gene in other A. baumannii strains. Other examples are the gene coding for the Ata adhesin (63) which was interrupted by an IS*Aba26* copy, and a gene encoding the O-antigen ligase TfpO normally involved in glycan decoration of capsule and proteins, which was interrupted by an IS*Aba13* copy in NCIMB8209 (64, 65). We then hypothesized that the lack of this group of genes could have impacted traits such as biofilm and pellicle formation as well as motility. To evaluate this hypothesis, the capacity of NCIMB8209 to form pellicle and biofilm when grown overnight in rich medium was compared to that of DSM30011, which was found to represent a high-biofilm/biopellicle producer (45). This assay indicated that NCIMB8209 was neither capable of forming pellicle (Fig. S4A) nor attaching to glass surfaces (Fig. S4B), supporting the above prediction that the ability to form biofilm/biopellicles is severely compromised in this strain. Furthermore, motility assays on semisolid medium showed that the ability of NCIMB8209 to perform surface-associated motility was highly reduced (Fig. S4B), again in sharp contrast to DSM30011 (46).

Another gene absent in strain NCIMB8209 is *cpaA* (Table S2), which encodes the surface-exposed metallopeptidase Cpa endowed with the ability to cleave fibrinogen and coagulation factor XII, and thus proposed to deregulate blood coagulation (66, 67). CpaA represents a substrate of the type II secretion system (68), and it has been proposed as a bona fide A. baumannii virulence factor (69). Therefore, its conspicuous absence in NCIMB8209 (Table S2) and also in its companion DSM30011, as well as in the clinical strains ATCC 17978 and ATCC 19606 isolated by the middle of last century (11) supports previous proposals that this gene was recently acquired by A. baumannii by horizontal gene transfer (68).

### Genetic features contributing to NCIMB8209 environmental adaptation. (i) Gene acquisition by lateral gene transfer.

Our genomic comparative analysis and subsequent BLASTN-based homology search (see Materials and Methods) indicated that 77 CDSs (59 CDSs located in the chromosome and the extra 18 in the plasmid) are unique to strain NCIMB8209 (Table S4). From the 59 unique chromosomal genes, 38 were located within different prophage and GI regions and showed no significant hits in databases. A BLAST search against the NCBI Protein database using as the query the amino acid sequences of the remaining 21 chromosomal CDSs revealed that 20 of them had significant best hits with proteins found in the database, 16 of them (i.e., 80%) located in species of the Acinetobacter genus (12 of them in A. baumannii) with potential roles in transport, motility, transcriptional regulation, and lipopolysaccharide synthesis (see Table S4 for details). Notably, four CDSs related to capsule synthesis (see below) were best affiliated with homologs located either in species of different orders among the class *Gammaproteobacteria* to which Acinetobacter belongs, including the *Alteromonodales* (*Alteromonas* sp.) and the *Enterobacteriales* (*Yersinia* sp.), and also to a different class (*Azoarcus* sp., *Betaproteobacteria*) or even to a different phylum (*Vitellibacter* sp., *Bacteroidetes/Chlorobi*) (Table S4). This indicated a remarkable ability of A. baumannii NCIMB8209 to co-opt genes from both phylogenetically related and distant species as the result of horizontal gene transfer.

### (ii) NCIMB8209 carries a novel K locus.

Among idiosyncratic features of strain NCIMB8209 worth noting, we found differences in content and organization of the genes linked to the production of the K capsule. The K locus identified in the NCIMB8209 genome (Table S2) displayed a gene organization that resembles the polysaccharide gene cluster PSgc6 reported for other clinical A. baumannii strains (70), with the exception of the *wafQRST* cluster which is missing in NCIMB8209. Furthermore, three extra genes were found in this novel K locus, namely, C4X49_00290 encoding an O-acetylase (absent in other members of the Acinetobacter genus), C4X49_00300 encoding a glycosyltransferase, and C4X49_00305 encoding a pyruvyl transferase (Table S2). Pyruvyl-capped *N*-acetyl-d-galactosamine (d-GalpNAcA) branches constitute rare structures described so far only in A. baumannii D78, a strain assigned to CC1 (71). The K-locus arrangement described above for strain NCIMB8209 was not found in other Acinetobacter strains by database searching, therefore revealing a previously unreported PSgc locus in A. baumannii. Of note, the *weeK* gene coding for an UDP-*N*-acetylglucosamine 4,6-dehydratase (involved in the biosynthesis of UDP-linked sugar precursors used for capsule synthesis) is also interrupted by an IS*Aba44* copy in NCIMB8209 (C4X49_00335-C4X49_00350, Table S2).

Strain NCIMB8209 shares with DSM30011 a similar gene locus involved in the synthesis of outer core polysaccharides (OC) of the lipid A core moiety, with the exception of an additional gene encoding a glycosyltransferase (C4X49_15205). However, this gene is annotated as a pseudogene and is probably nonfunctional (Table S2). As previously described for strain DSM30011 (11), this cluster includes the *rmlBDAC* genes (Table S2) responsible for the biosynthesis of dTDP-l-rhamnose (70, 72). The observed genetic organization of the OC locus in NCIMB8209 corresponds to OCL6 according to the classification proposed by Kenyon and collaborators (73).

### (iii) Mechanisms of resistance to toxic compounds.

Strain NCIMB8209 also contains many gene clusters encoding systems involved in the resistance to toxic compounds. Some of these clusters are scattered throughout the genome, while others are concentrated in three regions. One of these regions is constituted by GI3 (Table 2) integrated next to the *dusA* gene (C4X49_02945). The *dusA* locus has been found to represent a common integration site for this kind of genetic island in A. baumannii (74, 75). Although GI3 (19.1 kb long) is shorter than homologous GIs carried by other A. baumannii strains (for instance, in strain DSM30011, this GI is 33 kb long), this island still includes genes for putative arsenate and heavy metal ion detoxification systems (*ars* and *czc* genes) and other genes involved in Fe ion transport (*feoAB*). Another case is GI6, which carries a cluster of genes encoding a putative copper ion detoxification system (C4X49_13800 to C4X49_13840, Table 2). More interestingly, GI6 also harbors a *mobA* gene (C4X49_13855) encoding a protein with a relaxase domain which might be responsible for its mobilization after excision from the genome (Table 2). This may suggest a plasmid origin for this GI, and it also opens the possibility that this gene could even mediate its mobilization by horizontal gene transfer after excision from the genome. The third cluster (C4X49_16225 to C4X49_16400) contains a *merR-merTPCAD* gene cluster (C4X49_16305 to C4X49_16300 to C4X49_16280) coding for a complete Hg ion detoxification system (76). Since there is no gene coding for an integrase nearby, this region was not considered a GI. Remarkably, however, it is flanked by several IS copies which might have contributed to its mobilization and integration in this locus.

Inspection of the NCIMB8209 genome also evidenced the presence of three putative catalase genes (C4X49_07525, C4X49_02120, and C4X49_17825). Catalases represent one of the main strategies evolved by cells to cope with the accumulation of reactive oxygen species (77). It is then noteworthy that the number of putative catalase proteins encoded by this strain is higher than that of the environmental strains DSM30011 (two proteins, NCBI:protein PNH13446.1 and PNH14300.1) and A. baylyi ADP1 (one protein, CAG67388.1), and similar to that found in the A. baumannii clinical strain ATCC 17978 (three proteins, ABO11814.2, ABO10867.2, and ABO13771.2). Moreover, a comparative analysis of the tolerance of the above strains to strong oxidants, as judged by their survival when exposed to H_2_O_2_ (77), indicated a strong correlation between their catalase gene content and oxidative stress resistances (Fig. S6).

### (iv) Catabolic abilities.

Previous WGS analysis of the environmental A. baumannii DSM30011 strain predicted the presence in its genome of 28 gene clusters encoding many metabolic pathways involved in the utilization of a large variety of plant substances (11). The presence and organization of similar catabolic genes were also investigated in strain NCIMB8209, and despite some differences in the organization of catabolic loci between these two strains, 27 out of the 28 catabolic loci found in DSM30011 (11) were also present in this strain. The only exception was the salicylate/gentisate (*sal2*/*gen*) cluster, which was totally missing in NCIMB8209. In addition, the *betABI* locus present in both strains (previously thought to be a catabolic gene cluster [11, 44]) has been shown in A. baylyi to be involved in the synthesis rather than in the degradation of glycine betaine (78). In agreement, none of the A. baumannii strains tested including DSM30011, NCIMB8209, and ATCC 17978, nor A. baylyi, were capable of utilizing glycine betaine as the only carbon source for growth (Table S5).

From the 27 predicted catabolic loci shared between strains NCIMB8209 and DSM30011 mentioned above, 17 equivalent clusters are also found in the genome of A. baylyi and are involved in the degradation of plant substances and the recycling of plant material (44). These include loci such as *pca*, *qui*, *pob*, *hca*, *van*, and *ben*, involved in the degradation of aromatic acids and hydroxylated aromatic acids such as hydroxycinnamic acids constituting the building blocks of plant protective heteropolymers such as suberin (44, 79–81). These aromatic compounds are ultimately catabolized through the beta-ketoadipate pathway yielding Krebs cycle intermediary substrates, therefore allowing bacterial growth when used as the substrates (44). Our analysis of the ability of A. baumannii strains NCIMB8209 and DSM30011 to employ different compounds as the substrates for growth (Table S5) indicated that these two strains share with A. baylyi the ability to utilize many aromatic acids found in plants, including benzoate, 4-hydroxy-benzoate, 4-hydroxy-cinnamate, and shikimate, as the sole carbon sources. Moreover, the activity of the *mdc* pathway involved in the catabolism of dicarboxylic malonic acid, another plant-synthesized compound (44), was inferred from the growth observed by all A. baumannii strains tested in malonate as the only carbon source (Table S5). These observations are compatible with the isolation of A. baumannii strains NCIMB8209 and DSM30011 from an enriched consortium specialized in recycling resinous plant material (14). Remarkably still, all of the above-mentioned catabolic capabilities are also shared by A. baumannii clinical strains such as ATCC 17978 (Table S5 and data not shown).

Besides the above-described similarities with A. baylyi, strains NCIMB8209 and DSM30011 are endowed with some idiosyncratic catabolic clusters also related to the degradation of particular plant compounds. Among them we could mention the *paa* (phenylacetic acid [PAA]) and *liu* (leucine/isovalerate) clusters (11). The presence of a *paa* cluster in both NCIMB8209 and DSM30011, but not in A. baylyi, correlates with the capability of these A. baumannii strains to grow on PAA as the sole carbon source (Table S5). PAA is a plant auxin derived from the catabolism of phenylalanine (82, 83) endowed with substantial antimicrobial activity (84). PAA degradation by A. baumannii clinical strains has already been noted (82) (see also Table S5) and found to play an important role during A. baumannii infection by reducing the levels of this powerful phagocyte chemoattractant (83). Concerning the *liu* (leucine/isovalerate) catabolic cluster, evidence of its activity was obtained by the growth observed for NCIMB8209 and DSM30011 on l-leucine or isovalerate as the sole carbon sources (Table S5). In P. aeruginosa, the *liu* pathway complements the *atu* pathway responsible for the degradation of acyclic terpenes produced by plants in response to phytopathogens (85), and a similar situation may also occur in NCIMB8209 and DSM30011 (11). It follows that these A. baumannii strains have the capability not only to participate in the degradation of many plant aromatic compounds, but they are also endowed with the additional ability to degrade compounds produced by plants in response to stress situations, including the attack of phytopathogens and phytophagous insects (79–81).

Besides the above-described similarities at both the genomic level and metabolic capabilities between strains DSM30011 and NCIMB8209, a differential capacity for the utilization of the basic amino acids arginine and ornithine was observed between these two strains (Table S5). This may be explained by the differential presence in DSM30011 of a 10.5-kb fragment containing a *gdh-ascC-astA-astD-astB-astE* gene cluster encoding a complete arginine succinyltransferase (AST) pathway responsible for the catabolism of these basic amino acids (86), which was missing in NCIMB8209 (Fig. 1). Instead, a region of 27.6 kb bordered by IS*1008*/IS*Our* remnants and encompassing putative ion mercury detoxification genes was found in NCIMB8209, which has in turn been heavily impacted by several ISs of different types (Table S3). It is worth noting in the above context that the chromosomal region adjacent to *astA* in A. baumannii CC2 strains is also an integration site for different mobile elements such as AbGRI2-type resistance islands among others, which have provoked different rearrangements in their vicinity including various deletions (10, 53). This has resulted in some CC2 strains in which a AbGRI2-type element is found adjacent to a complete *ast* gene cluster, and other strains in which the *ast* genes have been completely deleted (10, 53; also data not shown). It has been shown in P. aeruginosa that the *N*-succinyl transferase AstA, the first enzyme of the AST pathway, is able to use both l-arginine and l-ornithine as the substrates (87), and a similar substrate specificity may also occur for A. baumannii AstA. Furthermore, genes coding for a putrescine importer (*puuP*) and a gamma-aminobutyraldehyde dehydrogenase (*patD*), the latter part of the transaminase pathway of putrescine degradation (86), were identified in DSM30011 (51% and 48% identity with the corresponding proteins from Escherichia coli strain K-12 substrain MG1655; accession numbers AAC74378.2 and AAC74526.1, respectively) but not in NCIMB8209. The latter observations might also explain the inability of NCIMB8209 to grow on putrescine, compared to the other A. baumannii strains tested (Table S12). In any case, the observed phenotypic profiles suggest a narrowing of NCIMB8209 substrate utilization capabilities when basic amino acids and polyamines in particular are considered, which suggests an adaptation of this strain to a specific niche.

### Conclusions.

A. baumannii NCIMB8209 represents to our knowledge the second reported environmental A. baumannii strain, isolated from a desert plant source at the onset (or even before) the massive introduction of antimicrobials to treat infections (11). In concordance, and similarly to its companion strain DSM30011, NCIMB8209 showed general susceptibility to most clinically employed antimicrobials, including folate pathway inhibitors (this work and reference 11). As expected from their common plant source and retting enrichment before isolation in media containing guayule resins as the substrates for growth (14), both A. baumannii strains share the ability to degrade a number of substances produced by plants, including many hydroxylated aromatic acids constituting the building blocks of plant protective hydrophobic heteropolymers and also repellents against predators (Table S5). However, WGS and subsequent phylogenetic and comparative genome analysis, as well as different biochemical studies conducted in this work, indicated significant differences between these two environmental strains. First, compared to DSM30011, NCIMB8209 has undergone a significant genome reduction and lacks many genetic clusters encoding components involved in defense mechanisms against other biological competitors such as the CRISPR-cas complex, T6SS, and two-partner systems. Moreover, and although NCIMB8209 contains most genes associated with persistence and virulence in A. baumannii clinical strains, many genes encoding components of surface structures are interrupted by IS elements whose relatively high number and variability impacted heavily on the NCIMB8209 genome. Among IS-interrupted genes, we found those encoding pili components, the O-antigen ligase TfpO, a biosynthetic route for surfactants compounds, Bap and Ata adhesins, a RTX-toxin, etc. (Table S3). Comparative biofilm and pellicle production, as well as motility assays, yielded evidence for the fact that NCIM8209 is severely compromised in these pathogenicity-associated traits (Fig. S4). Altogether, these observations can explain the low relative virulence potential observed for NCIM8209 on the G. mellonella and C. elegans infection models (Fig. S3).

Loss of T6SS genes has been observed in a number of A. baumannii clinical strains causing infections, suggesting that this system is not required once A. baumannii invades its host (53, 88, 89). Moreover, the absence of T6SS has been linked with higher chances of evasion of A. baumannii from the host immune system (90, 91). The situation described above for strain NCIMB8209 therefore resembles that reported for A. baumannii SDF isolated from a human body louse (24), which has undergone extensive genome reductions and rearrangements mediated by ISs (24). Also, albeit more moderately, loss of T6SS and IS-mediated inactivation of genes encoding surface structures was also observed in A. baumannii D1279779, a community-acquired strain isolated from the bacteremic infection of an indigenous Australian (26).

It is tempting to speculate that the changes observed in A. baumannii NCIMB8209 are also related to its adaptation to a particular niche. In this context, several reports have shown the profound associations existing between different bacterial groups, including many members of the Acinetobacter genus with a number of insects feeding on plants (16–22). Many of these bacterial species are located in the guts of their insect hosts conducting mutualistic or symbiotic associations based on nutrition/protection relationships (16, 18–20, 92, 93). The bacterial counterpart thus degrades toxic compounds for the insect of the plant diet, and the insect host in return provides a stable environment, supply, or resources, and a vector for the rapid spreading and inoculation into fresh plant tissues. For those bacterial species displaying low pathogenic potential, selective pressure eventually favors more stable relationships with concomitant structural and metabolic changes (93). A general trend thus observed for Gram-negative species with these characteristics is genome reduction with the loss or modification of surface-exposed molecules, thus reducing interactions with the innate immune system of their hosts which may trigger defensive responses and elimination (92, 93). This situation can certainly be applied to NCIMB8209, as extensively detailed above. In this context, we note the relative high tolerance of this strain to pro-oxidants such as H_2_O_2_ (Fig. S6), which correlates with the identification of three catalase genes in its genome (94). The innate immune system of insects closely resembles that of vertebrates at both the molecular and cellular levels (92). Thus, while the cellular immune response in vertebrates is mediated by professional phagocytes, in insects, this function is conducted by phagocytic cells known as hemocytes (92). Since phagocytic cells use oxidative burst as a common strategy to counteract pathogens (92, 95), it is then possible that the selection of a higher antioxidant ability in NCIMB8209 led to an increased tolerance to oxidative stress and therefore increased survival in an insect niche. Of note, recent evidence indicates that A. baumannii strains with high catalase production are more resistant against intracellular killing by macrophages (96).

Our observations also provide further clues on the high genomic plasticity of A. baumannii as a species (6), underscoring the fact that this highly advantageous feature in adaptive terms is not exclusive to A. baumannii strains of clinical origin only. In summary, WGS analysis and complementary phenotypic characterization of A. baumannii strains of environmental origin such as DSM30011 and NCIMB8209 provided important clues on the genomic content and diversity of this species before the strong selection pressure associated with the current antibiotic era, and suggested potential niches of this species outside the clinical setting.

## MATERIALS AND METHODS

### Bacterial strains and growth conditions.

Acinetobacter sp. strain NCIMB8209 was obtained from the National Collection of Industrial Food and Marine Bacteria, Aberdeen, Scotland. For details on the original isolation and different denominations assigned to this strain by various collections, see our previous publication (11) under “Tracing DSM30011 origins.”

The use of carbon or carbon and nitrogen sources by the A. baumannii strain NCIMB8209, DSM30011, or ATCC 17978 was conducted on solid BM2 minimal medium [62 mM potassium phosphate (pH 7.0), 7 mM (NH_4_)_2_SO_4_, 0.5 mM MgSO_4_, 10 μM FeSO_4_] supplemented with 1.5% Difco bacteriological agar and 0.2% (wt/vol) of the tested substrates (97). The plates were incubated at 30°C for 48 to 96 h before colony growth was inspected. Other bacterial strains such as Acinetobacter baylyi ADP1 (44) and Pseudomonas aeruginosa PAO1 (98) were also included in these tests as controls for the utilization of particular compounds.

Plates containing 0.3% agarose, 10 g/liter tryptone, and 5 g/liter NaCl were used as a tool to detect cell motility on a semisolid surface. The plates were inoculated on the surface with bacteria lifted from overnight LB agar cultures using flat-ended sterile wooden sticks or depositing 10 μl of LB cultures grown to an optical density at 600 nm (OD_600_) of 0.1. Plates were incubated for 30 h at 30°C in the dark before inspection and recording.

Tolerance to H_2_O_2_ was performed as previously described (77). Briefly, bacterial cultures were collected when they reached an OD_600_ of 0.6 and subjected to serial dilutions. Aliquots of 10 μl were then loaded onto LB agar plates, supplemented with 400 μM H_2_O_2_, and incubated for 30 h at 30°C before inspection.

### NCIMB8209 genome sequencing and annotation.

NCIMB8209 DNA was isolated using a commercial kit (Wizard Genomic DNA purification kit; Promega), following the manufacturer’s instructions. The genomic sequence was obtained using a hybrid strategy combining PacBio Sequel (MrDNALab, Shallowater, TX, USA) and Illumina MiSeq (single-end reads, University of Chicago genomic facilities) methods. A total of 3,238,476,791 reads were generated with the PacBio Sequel approach (depth of coverage ∼834×), which were subjected to quality assessment prior to *de novo* genome assembly using Falcon (99) followed by further polishment by the Arrow algorithm. In turn, the sequence data generated by the Illumina strategy were assembled into contigs using Spades version 3.0.11 (100), and used to close the gaps left by the PacBio Sequel strategy. The replication origin (*oriC*) was predicted with OriFinder (101). The final assembled NCIMB8209 chromosome was annotated using the pipeline available at NCBI and deposited in DDBJ/ENA/GenBank under accession number CP028138. In turn, the plasmid sequences derived from the above sequencing data were deposited in the GenBank nucleotide sequence database under accession number CP028139.

The presence of IS elements in the NCIMB8209 genome was determined using IS Finder version 2.0 (102) (https://www-is.biotoul.fr/). Novel ISs were deposited into the ISSaga database (54). Putative prophage sequences were identified by PHASTER (103, 104). Antimicrobial resistance genes were detected with ResFinder 3.2 (https://cge.cbs.dtu.dk/services/ResFinder/) (105). Catabolic clusters present in the NCIMB8209 genome were searched by a BLASTN approach (106) using as queries the catabolic genes previously described for the DSM30011 strain (11).

### Protein family analyses and phylogenomic calculations.

The proteomes of the 126 Acinetobacter strains analyzed in this work, which included 99 A. baumannii strains other than NCIMB8209 and DSM30011, were first retrieved from the NCBI ftp database and gathered into a local database together with the NCIMB8209 proteome. Families of homologous proteins were clustered with PGAP version 1.2.1 by using the gene family (GF) method (107). Briefly, the BLASTALL tool was executed among all mixed protein sequences, and the filtered BLAST result was clustered by the MCL algorithm. For each protein pair of the same cluster, the global match region and identity were set to be equal or higher than 70% of the longer sequence. This analysis allowed us to assemble 27,367 protein families among these genomes, from which 6,383 corresponded to protein families presenting a single representative in all Acinetobacter genomes analyzed. Regarding strain NCIMB8209 (3,596 predicted total CDSs), 3,551 protein families contained at least one representative sequence in this strain, including 79 families that contained more than one.

Maximum likelihood (ML) phylogenetic trees were then constructed using these data. For each protein family, the corresponding nucleotide sequences were retrieved, individually aligned using ClustalW2 (108), and trimmed using GBlock 0.91b (109). The resulting alignments were combined to build a large supermatrix (362,285 nucleotide positions) using the Python3 package AMAS 0.98 (110). Finally, the evolutionary history of the strains was inferred as implemented by Espariz et al. (111) using the RAxML software (112) and the general tree reversible (GTR) substitution model with gamma distribution. The individual parameters for the model were estimated and optimized for each concatenated gene as indicated by Stamatakis (112). Reliability of the inferred tree was tested by bootstrapping with 100 repetitions.

In order to depurate the list of protein families specific for strain NCIMB8209 according to PGAP predictions, their coding genes were used as queries in a BLASTN sequence similarity-based comparison (106) against all 126 Acinetobacter genomes present in our local database. Coverage and identity thresholds of 70% and an E-value cutoff of 1E−30 were used for these calculations. Those CDSs for which a significant hit was found were then deleted from the list.

### Sequence typing.

Assignment of sequence types (ST) for strain NCIMB8209 was done using the housekeeping genes *cpn60*, *gltA*, *gpi*, *gyrB*, *recA*, and *rpoD* (Oxford scheme) (29) or *cpn60*, *fusA*, *gltA*, *pyrG*, *recA*, *rplB*, and *rpoD* (Pasteur scheme) (3). For details, see the A. baumannii MLST Databases website (http://pubmlst.org/abaumannii/) (113).

### *gdhB* gene detection by PCR.

PCRs were carried out following standard protocols, using the primer pair GHDB_1F (GCTACTTTTATGCAACAGAGC)/GHDB775R (GTTGAGTTGGCGTATGTTGTGC) for *ghdB* detection and the degenerate primer pair 16S RDNAF (AGAGTTTGATCHTGGYTYAGA)/16S_RDNAR (ACGGYTACCTTGTTACGACTTC) for 16S rRNA gene detection.

### Antimicrobial susceptibility testing.

The general antimicrobial susceptibility of strain NCIMB8209 was evaluated using the Vitek 2 System (bioMérieux) following criteria recommended by the Clinical and Laboratory Standards Institute (CLSI) (114). Susceptibility tests to tetracycline, chloramphenicol, and macrolides such as azithromycin and erythromycin were done separately by disk assays on Mueller-Hinton agar (MHA) following CLSI protocols. In short, NCIMB8209 cells were grown overnight at 37°C, resuspended in LB broth to a turbidity of 0.5 McFarland units, and spread on the surfaces of MHA-containing petri plates. Antibiotic disks were then carefully deposited at the center of the agar surface, and the plates were incubated at 37°C for 16 h before measuring the diameter of the corresponding growth inhibition zones.

### Bacterial competition assays.

The different A. baumannii and E. coli strains used in competition assays were grown overnight in 2 ml of L-broth (10 g/liter tryptone, 5 g/liter yeast extract, and 0.5 g/liter NaCl) medium. Cultures were then diluted in L-broth medium to an absorbance at 600 nm (Abs600) of 0.1. The cells were mixed in a 10:1 ratio (predator/prey). Aliquots of 20 μl of these cell mixtures were laid on the surface of L-broth medium supplemented with 1.5% agar and incubated at 37°C for 4 h. The bacterial spot on the agar surface was subsequently removed and vigorously resuspended in an Eppendorf tube with 500 μl of PBS. The mixtures were serially diluted 1:10 in PBS using a 96-well polystyrene microtiter plate and then plated onto solid selective media for colony counting. For E. coli DH5α, selective medium containing 20 μg/ml nalidixic acid was used. For Acinetobacter strains, selective media contained either 30 μg/ml gentamicin or 75 μg/ml rifampicin were employed as indicated. For all bacterial competition experiments, one representative image of the growth results obtained in selective medium is shown.

### Hcp secretion analysis.

Experiments were performed by the method of Repizo et al. (45). Briefly, the A. baumannii strains analyzed for Hcp secretion were inoculated in 50 ml L-broth at an Abs600 of 0.08, and incubated at 37°C until the Abs600 reached a value of ∼1.0. Culture supernatants were obtained by centrifugation at 4,000 × *g*, followed by filtration using 0.22-μm filters. Secreted proteins present in the supernatants were then concentrated 50-fold using Amicon Ultracel 3K centrifuge filters following the instructions of the manufacturer and subjected to a wash step with 40 mM Tris (pH 8), 200 mM NaCl, and 5% glycerol before being analyzed by 18% sodium dodecyl sulfate-polyacrylamide gel electrophoresis (SDS-PAGE).

### Biofilm assays.

Biofilm formation was qualitatively determined by measuring the adhesion of bacteria to the surfaces of glass tubes. A. baumannii strains were statically grown overnight in 2 ml of L-broth medium. The spent liquid medium was discarded, and the tubes were rinsed twice with water before adding a solution of 1% crystal violet. After 15 min, the dye solution was discarded, and the tubes were rinsed twice with water before inspection. All assays were done at least three times using fresh samples each time.

### A. baumannii virulence assays using Caenorhabditis elegans nematode and Galleria mellonella larvae model systems.

Virulence assays of the different A. baumannii strains tested here were evaluated by using two different model systems: G. mellonella moth larvae (42) and the nematode C. elegans (43). G. mellonella larvae were purchased from Knutson’s Live Bait (Brooklyn, MI) and were used the day after arrival. Groups of 20 randomly picked larvae were used for each assay condition. The different A. baumannii strains tested were grown overnight in LB and then diluted with phosphate-buffered saline (PBS) to obtain the CFU titers indicated in the corresponding figure legends, which were verified by colony counts on L-broth agar (LBA) for all inocula. A Hamilton microliter syringe was used to inject 10 μl of the bacterial suspensions into the hemolymph of each larva via the second to the last left proleg. For a control, one group of G. mellonella larvae was injected with 10 μl of PBS. After injection, the larvae were incubated in plastic plates at 37°C, and the numbers of dead individuals were scored regularly.

For C. elegans survival experiments, the N2 Bristol (wild type) was used. Gravid hermaphrodites were bleached, and the resulting eggs were washed using standard protocols (115). Nematodes arrested in larval stage 1 (L1) were then transferred onto NGM plates seeded with fresh E. coli OP50 and grown at 20°C until nematodes reached larval stage 4 (L4). For virulence assays, A. baumannii and E. coli OP50 (control) were grown overnight at 37°C in L-broth medium. The stationary culture was then diluted to 10^7^ total CFU, and 50-μl portions were seeded onto 60-mm nematode growth medium (NGM) plates. Groups of L4 worms were then transferred to the plates seeded with A. baumannii and deposited on the center of the bacterial spot. Assays were performed in duplicates. The nematodes were maintained at 20°C, transferred to fresh plates every 48 h, and monitored daily. Worms that did not respond to stimulation by touch were scored as dead.

Survival curves were plotted using PRISM software, and comparisons in survival were calculated using the log rank Mantel-Cox test and Gehan-Breslow-Wilcoxon test.

### Data availability.

The final assembled NCIMB8209 chromosome was annotated using the pipeline available at NCBI and deposited in DDBJ/ENA/GenBank under accession number CP028138. In turn, the plasmid sequences derived from the above sequencing data were deposited in the GenBank nucleotide sequence database under accession number CP028139.
